# Fruit and vegetable consumption and metabolic syndrome in Chinese adults: a cross-sectional study

**DOI:** 10.3389/fnut.2026.1852298

**Published:** 2026-06-30

**Authors:** Bing Wang, Xiao-Yan Qian, Xiao-Fang Chen, Ying-Jie Wang, Hui Zhou, Li-Qiang Qin, Guang-Fang Shao, Khemayanto Hidayat

**Affiliations:** 1 Department of Nutrition and Food Hygiene, School of Public Health, Soochow University, Suzhou, China; 2 Suzhou Industrial Park Centers for Disease Control and Prevention, Suzhou, China; 3 Jianhu County Center for Disease Control and Prevention, Jianhu, China

**Keywords:** central obesity, fruit & vegetable intake, hyperglycemia, hypertension, hypertriglyceridemia, metabolic syndrome

## Abstract

**Background:**

Evidence suggests inverse associations between fruit and vegetable intakes and metabolic syndrome (MetS), but data from Chinese populations remain limited, particularly regarding component-specific associations and whether associations vary according to concurrent intake of other major food groups.

**Objective:**

To examine associations of fruit and vegetable intakes with MetS and its individual components, and to explore whether these associations vary across co-consumption patterns of other major food groups.

**Materials and methods:**

This community-based cross-sectional study included 5,107 adults from Suzhou, China. Dietary intake was assessed using a food frequency questionnaire. Multivariable logistic regression models were used to estimate adjusted odds ratios (ORs) and 95% confidence intervals (CIs). Joint analyses evaluated combinations of fruit or vegetable intake with other major food groups.

**Results:**

Higher fruit and vegetable intakes were associated with lower odds of MetS. Comparing the highest with the lowest quartile, adjusted ORs were 0.82 (95% CI 0.69–0.97; *p*-trend = 0.02) for fruit and 0.84 (95% CI 0.70–0.99; *p*-trend = 0.03) for vegetables. In continuous analyses, ORs were 0.90 (95% CI 0.85–0.95) per 100 g/day increase in fruit intake and 0.91 (95% CI 0.85–0.98) per 200 g/day increase in vegetable intake. Fruit intake was inversely associated with multiple MetS components, whereas vegetable intake showed more selective associations. Joint analyses suggested that higher fruit or vegetable intake combined with lower red meat intake was associated with lower odds of MetS and several of its components.

**Conclusion:**

In this community-based sample of Chinese adults, higher fruit and vegetable intakes were associated with lower odds of MetS, with fruit showing broader inverse associations across individual components than vegetables. Exploratory joint analyses suggested that these associations varied according to co-consumption patterns of other major food groups, particularly red meat intake. Prospective studies are needed to clarify temporality and causality.

## Introduction

Metabolic syndrome (MetS), characterized by a clustering of central obesity, dyslipidemia, elevated blood pressure, and impaired glucose metabolism, represents a major public health challenge worldwide. Individuals with MetS are at substantially increased risk of type 2 diabetes, cardiovascular disease, and premature mortality (1). The global prevalence of MetS has increased steadily over recent decades, underscoring its growing public health impact across diverse populations (2–4). In China, the prevalence of MetS rose markedly from 8.8% in 1991–1995 to 29.3% in 2011–2015, with a more pronounced increase among women (from 7.9 to 30.7%) than among men (from 9.4 to 27.2%) (4). Given this rapid rise, identifying modifiable dietary factors associated with MetS and its individual components may help inform population-level prevention and intervention strategies.

Adequate consumption of fruits and vegetables has been associated with favorable cardiometabolic outcomes, potentially owing to their high content of dietary fiber and phytochemicals (5–8). Consistent with this evidence, the Chinese Dietary Guidelines recommend that adults consume 200–350 g of fruit and 300–500 g of vegetables per day to promote optimal health (9). However, adherence to these recommendations remains suboptimal, particularly for fruit intake (10). At the same time, dietary patterns in China have undergone substantial transitions in recent decades, characterized by reduced consumption of traditional plant-based foods and increased intake of animal-source foods, particularly red meat, alongside increasingly energy-dense diets in urban populations. These dietary changes have coincided with a rising burden of obesity and other cardiometabolic disorders in China (11). Since fruits and vegetables are consumed within broader dietary patterns rather than in isolation, such shifts in overall dietary composition may complicate the interpretation of associations between fruit and vegetable intakes and metabolic health outcomes.

In habitual Chinese diets, higher intake of fruits and vegetables often co-occurs with varying levels of consumption of other major food groups, including red meat, poultry, fish, soy products, dairy, and nuts. Prior analyses conducted in this same community-based population have demonstrated that these food groups exhibit distinct—and sometimes opposing—associations with MetS and its individual components (12–14). Such dietary interrelationships raise the possibility that observed associations between fruit and vegetable intake and MetS may depend, at least in part, on the broader dietary context in which they are consumed.

Evidence from observational studies, largely conducted in Western populations, suggests that higher fruit and vegetable intakes are associated with lower odds of MetS (15, 16). However, data from Chinese populations remain relatively limited (17–20). Existing studies from mainland China and Taiwan have typically examined fruit and/or vegetable intake as secondary exposures within broader lifestyle or risk factor analyses, rather than as primary exposures of interest. Most prior investigations have focused exclusively on MetS as a composite outcome and have not examined its individual components separately. This distinction is important because associations with MetS may be driven by specific components—such as central obesity, hypertriglyceridemia, hypertension, or impaired glucose regulation—that differ in their relationships with fruit and vegetable intake. Furthermore, limited adjustment for other dietary factors and the use of broad intake categories have constrained efforts to clarify independent and component-specific associations in Chinese adults.

Therefore, using data from a community-based sample of Chinese adults, the present study aimed to: (1) examine associations of fruit and vegetable intakes—modeled both categorically and continuously—with MetS and its individual components; (2) evaluate potential heterogeneity of these associations across key population subgroups; and (3) explore how these associations vary across co-consumption patterns of other major food groups. By addressing both component-specific outcomes and dietary co-consumption patterns, this study seeks to provide a more comprehensive understanding of the relationship between fruit and vegetable intakes and metabolic health in Chinese adults.

## Materials and methods

### Study design and population

This cross-sectional analysis was conducted using data from a community-based health survey carried out between July 2013 and November 2014 in four residential communities in the Suzhou Industrial Park, Suzhou City, Jiangsu Province, China. All individuals aged 18 years or older who were registered residents of the selected communities were invited to participate through public announcements and outreach at local community health service centers. Among 7,866 participants who completed the baseline survey and provided written informed consent, individuals were included in the present analysis if they had complete information on fruit and vegetable intakes and all components of MetS. No additional inclusion or exclusion criteria were applied. After exclusions for missing exposure or outcome data, the final analytic sample comprised 5,107 participants. All data collection procedures, including interviews, physical examinations, and biological sample collection, were conducted by trained personnel using standardized protocols. The study protocol was approved by the Ethics Committee of Soochow University (approval number: ECSU-2010-002) and was conducted in accordance with the principles of the Declaration of Helsinki.

### Dietary assessments

Habitual dietary intake over the previous year was assessed using a structured, interviewer-administered food frequency questionnaire (FFQ). Participants reported the frequency and usual portion size of major food groups, including fruits, vegetables, soy, red meat, poultry, fish, dairy, nuts, and salted vegetables. Salted vegetables were assessed as a separate food group and were not included in the primary vegetable intake exposure. Participants selected one of five frequency categories (never, yearly, monthly, weekly, or daily) and reported usual portion sizes using standard household measures [斤 (jin), 两 (liang), or mL]. Average daily intake (g/day) was calculated by multiplying the reported frequency by the portion size and converting the result to grams using standard food composition tables. The FFQ was designed primarily to rank habitual intake of major food groups rather than estimate nutrient or total energy intake; therefore, detailed information on total energy intake and cooking methods was not available. In addition, the FFQ has not been formally validated against repeated dietary recalls or biomarkers in this population.

### Anthropometric and biochemical measurements

Fasting venous blood samples were collected after an overnight fast of 10–12 h. Plasma concentrations of fasting glucose, total cholesterol, triglycerides, high-density lipoprotein cholesterol (HDL-C), and low-density lipoprotein cholesterol (LDL-C) were measured using an automated biochemical analyzer (Olympus AU640, Kobe, Japan). Blood pressure was measured with participants seated using a calibrated mercury sphygmomanometer (Shanghai Zhangdong Med-Tech Ltd., Shanghai, China). Two measurements were taken after at least 5 min of rest, and the average was used for analysis. Anthropometric measurements were obtained by trained staff following standardized procedures. Body weight was measured to the nearest 0.1 kg, and height and waist circumference were measured to the nearest 0.1 cm. Body mass index (BMI) was calculated as weight (kg) divided by height squared (m^2^).

### Assessment of covariates

Sociodemographic and lifestyle characteristics were collected through structured interviews. Covariates included age, sex, educational attainment (<high school, high school/vocational, ≥college), smoking status (never, former, current), alcohol consumption (0, 1–3, or >3 times/week), physical activity level (self-reported), sleep duration (hours/day), and television watching time (hours/day).

### Definition of metabolic syndrome

MetS was defined according to the Joint Interim Statement (JIS) criteria (1). Participants were classified as having MetS if they met three or more of the following five criteria:

1. Elevated waist circumference: ≥90 cm for men or ≥80 cm for women (Asian-specific cut-offs);
2. Elevated triglycerides: ≥150 mg/dL (1.7 mmol/L);
3. Reduced HDL-C: <40 mg/dL (1.0 mmol/L) in men or <50 mg/dL (1.3 mmol/L) in women;
4. Elevated blood pressure: systolic blood pressure ≥130 mmHg or diastolic blood pressure ≥85 mmHg;
5. Elevated fasting glucose: ≥100 mg/dL (5.6 mmol/L).

The use of medications for diabetes, dyslipidemia, or hypertension was also considered when classifying the corresponding components.

### Statistical analysis

Participants were categorized into quartiles according to their average daily intakes of fruits and vegetables. Differences in participant characteristics across quartiles were assessed using one-way analysis of variance (ANOVA) for continuous variables and chi-square tests for categorical variables.

Multivariable logistic regression models were used to estimate odds ratios (ORs) and 95% confidence intervals (CIs) for MetS and its individual components across quartiles of fruit and vegetable intakes, with the lowest quartile serving as the reference category. Given the cross-sectional design, ORs should be interpreted as prevalence ORs. Linear trends across quartiles were evaluated by modeling the median intake of each quartile as a continuous variable.

Fruit and vegetable intakes were analyzed both as categorical variables (quartiles) and as continuous variables, modeled per 100 g/day increase in fruit intake and per 200 g/day increase in vegetable intake to reflect meaningful differences in habitual consumption within the study population. Restricted cubic spline analyses were additionally performed to evaluate potential non-linear associations between fruit and vegetable intakes and MetS, using knots placed at the 10th, 50th, and 90th percentiles of the intake distributions. Tests for non-linearity were conducted by evaluating the statistical significance of the non-linear spline terms.

All multivariable models were adjusted for age, sex, educational level, smoking status, alcohol consumption, physical activity, body mass index (BMI), sleep duration, television watching time, and intake of other major food groups. Fruit and vegetable intakes were mutually adjusted in all relevant models.

Total energy intake was not included in the primary models because the FFQ was designed primarily to rank habitual intake of major food groups rather than estimate total energy intake, and detailed energy intake data were therefore unavailable.

Variance inflation factors were examined to assess potential multicollinearity among dietary variables included in the multivariable models, and no evidence of substantial multicollinearity was observed (all variance inflation factors <2.5).

Stratified analyses were conducted to examine whether associations differed by age (<55 vs. ≥55 years), sex, BMI (<25 vs. ≥25 kg/m^2^), and smoking status (never vs. former/current smokers). To preserve statistical power, subgroup analyses were performed with fruit and vegetable intakes modeled as continuous variables. Within each subgroup, ORs and 95% CIs were estimated for a 100 g/day increase in fruit intake and a 200 g/day increase in vegetable intake using multivariable logistic regression models, adjusted for the same covariates as in the primary analyses, with the stratifying variable excluded from the corresponding model. Statistical interaction was assessed by including multiplicative interaction terms between continuous fruit or vegetable intake and the stratifying variable in fully adjusted models, and *p* values for interaction were derived using Wald tests.

Joint analyses were conducted to examine whether associations of fruit and vegetable intake with metabolic outcomes varied across co-consumption patterns of other major food groups. These analyses were exploratory and do not represent formal tests of interaction or comprehensive dietary pattern analyses. Fruit, vegetables, red meat, poultry, fish, and soy were dichotomized into high- and low-intake categories based on the median intake in the study population. Because intakes of nuts and dairy were highly skewed, these food groups were categorized as non-consumers and consumers. Joint exposure categories were then created by combining fruit or vegetable intake with intake of red meat, poultry, fish, soy, nuts, or dairy. For each joint analysis, the reference category comprised participants with lower fruit or vegetable intake and lower intake of the corresponding food group; for nuts and dairy, the reference category comprised participants with lower fruit or vegetable intake and no consumption of the corresponding food group. Associations with metabolic outcomes were evaluated using multivariable logistic regression models. All joint models were adjusted for the same covariates as the primary analyses; when a food group was included in a joint exposure, it was excluded from the corresponding adjustment set to avoid over-adjustment from including the same exposure in both the model and the joint variable.

All statistical analyses were performed using SPSS version 20.0 (SPSS Inc., Chicago, IL, USA). All tests were two-sided, and *p* values <0.05 were considered statistically significant.

## Results

### Participant characteristics

Participant characteristics according to quartiles of fruit and vegetable intakes are shown in Table 1. Higher intakes of fruits and vegetables were associated with younger age, higher educational attainment, lower waist circumference, lower systolic and diastolic blood pressure, lower fasting glucose concentrations, higher HDL-C levels, lower prevalence of current smoking, and slightly longer sleep duration (all *p* ≤ 0.031).

**Table 1:** Characteristics of the study participants according to fruit or vegetable intake quartiles.

Characteristics	Fruits					Vegetables				
	Q1 (*n* = 1,278)	Q2 (*n* = 1,283)	Q3 (*n* = 1,279)	Q4 (*n* = 1,267)	*p*	Q1 (*n* = 1,280)	Q2 (*n* = 1,277)	Q3 (*n* = 1,285)	Q4 (*n* = 1,265)	*p*
Fruits (g/day)	15.65 ± 9.87	60.30 ± 18.42	147.96 ± 32.16	261.93 ± 74.51	<0.001	97.08 ± 38.66	100.69 ± 43.21	126.35 ± 51.14	135.82 ± 56.42	<0.001
Vegetables (g/day)	284.46 ± 86.72	302.05 ± 92.71	345.60 ± 104.43	362.59 ± 118.06	<0.001	152.25 ± 47.12	265.93 ± 69.76	391.48 ± 109.57	542.46 ± 158.22	<0.001
Demographic characteristics										
Age in years	56.95 ± 9.84	54.06 ± 9.55	53.08 ± 9.09	51.37 ± 9.37	<0.001	55.09 ± 10.54	54.05 ± 9.98	52.99 ± 8.94	54.33 ± 9.01	<0.001
Sex										
Men	573 (44.8)	525 (41)	557 (43.6)	629 (49.6)	<0.001	594 (46.4)	539 (42.2)	565 (44)	586 (46.3)	<0.001
Women	705 (55.2)	758 (59)	722 (56.4)	638 (50.4)		686 (53.6)	738 (57.8)	720 (56)	679 (53.7)	
Education										
<High school	1,218 (95.3)	1,193 (93)	1,176 (92)	1,164 (91.9)	0.002	1,225 (95.7)	1,188 (93)	1,184 (92.2)	1,154 (91.2)	0.003
High school/vocational	54 (4.2)	83 (6.5)	93 (7.2)	85 (6.7)		45 (3.5)	77 (6)	93 (7.2)	100 (7.9)	
≥College	6 (0.5)	7 (0.5)	10 (0.8)	18 (1.4)		10 (0.8)	12 (1)	8 (0.6)	11 (0.9)	
Behavioral characteristics										
Physical activity	35.41 ± 21.45	35.52 ± 22.67	36.34 ± 24.38	35.26 ± 22.62	0.148	34.69 ± 22.48	35.75 ± 23.65	36.88 ± 22.73	35.66 ± 22.57	0.227
Alcohol										
0/week	1,012 (79.2)	1,050 (81.8)	980 (76.6)	974 (76.9)	<0.001	1,004 (78.4)	1,050 (82.2)	975 (75.9)	968 (76.5)	<0.001
1-3/week	93 (7.3)	79 (6.2)	174 (13.6)	146 (11.5)		101 (7.9)	75 (5.9)	182 (14.2)	151 (11.9)	
>3/week	154 (12)	140 (10.9)	113 (8.8)	138 (10.9)		139 (10.9)	135 (10.6)	112 (8.7)	134 (10.6)	
Unknown	19 (1.5)	14 (1.1)	12 (1)	11 (0.9)		36 (2.8)	17 (1.3)	16 (1.2)	12 (1)	
Smoking										
Never	837 (65.5)	874 (68.1)	871 (68.1)	882 (69.7)	<0.001	936 (73.1)	854 (66.9)	846 (65.8)	828 (65.4)	<0.001
Former	36 (2.8)	31 (2.4)	34 (2.6)	36 (2.8)		31 (2.4)	37 (2.9)	32 (2.5)	37 (3.0)	
Current	361 (28.3)	356 (27.8)	341 (26.7)	332 (26.2)		303 (23.7)	364 (28.5)	369 (28.7)	354 (28.0)	
Unknown	44 (3.4)	22 (1.7)	33 (2.6)	17 (1.3)		10 (0.8)	22 (1.7)	38 (3.0)	46 (3.6)	
Sleep duration (hours/day)	7.22 ± 1.12	7.37 ± 0.99	7.36 ± 0.89	7.31 ± 0.96	<0.001	7.18 ± 1.09	7.35 ± 1.01	7.37 ± 0.89	7.30 ± 1.04	<0.001
Television watching (hours/day)	3.98 ± 2.99	3.07 ± 3.02	5.42 ± 2.55	5.19 ± 2.56	<0.001	4.53 ± 2.87	3.29 ± 3.00	5.53 ± 2.56	4.46 ± 2.79	<0.001
Dietary intake (g/day)										
Soy	23.70 ± 30.72	39.64 ± 43.85	47.20 ± 58.60	93.70 ± 69.08	<0.001	37.01 ± 56.93	33.35 ± 35.14	87.49 ± 59.97	37.90 ± 70.14	<0.001
Red meat	38.95 ± 41.30	53.13 ± 41.30	61.38 ± 45.95	81.05 ± 47.04	<0.001	46.04 ± 32.45	47.51 ± 38.32	78.85 ± 48.33	59.76 ± 59.71	<0.001
Poultry	26.62 ± 25.21	43.39 ± 39.12	57.78 ± 53.97	77.11 ± 56.42	<0.001	39.43 ± 32.30	35.16 ± 36.17	75.92 ± 51.73	54.01 ± 63.04	<0.001
Fish	42.73 ± 40.79	61.11 ± 44.17	77.12 ± 71.64	98.29 ± 70.13	<0.001	47.16 ± 37.18	61.07 ± 58.43	89.82 ± 55.43	74.50 ± 82.93	<0.001
Dairy	15.82 ± 48.02	17.31 ± 57.33	18.92 ± 65.13	29.30 ± 91.45	<0.001	15.18 ± 50.17	20.73 ± 64.06	18.52 ± 56.83	26.18 ± 98.70	0.008
Nuts	6.36 ± 10.05	5.78 ± 18.17	9.93 ± 16.76	10.11 ± 22.29	<0.001	7.48 ± 26.30	10.02 ± 14.15	6.10 ± 15.47	7.43 ± 14.02	<0.001
Salted vegetables	16.21 ± 16.69	20.89 ± 21.13	18.35 ± 29.70	33.59 ± 37.89	<0.001	13.96 ± 17.89	17.92 ± 20.37	32.04 ± 36.96	24.15 ± 28.66	<0.001
Cardiometabolic markers										
Body weight (kg)	60.93 ± 10.17	61.74 ± 9.78	61.51 ± 10.06	61.53 ± 9.74	0.189	61.61 ± 9.91	62.34 ± 10.11	60.92 ± 9.76	60.98 ± 9.96	0.008
BMI (kg/m^2^)	23.81 ± 3.18	23.93 ± 3.06	23.87 ± 3.08	23.69 ± 3.04	0.262	24.10 ± 3.11	23.88 ± 3.09	23.70 ± 3.08	23.69 ± 3.09	0.018
Waist circumference (cm)	82.14 ± 8.41	81.64 ± 8.47	81.47 ± 8.73	81.12 ± 8.28	0.031	82.10 ± 8.49	82.02 ± 8.43	81.37 ± 8.39	81.23 ± 8.56	0.013
Triglyceride (mmol/L)	1.59 ± 1.21	1.60 ± 1.19	1.55 ± 1.12	1.50 ± 1.24	0.183	1.62 ± 1.29	1.57 ± 1.24	1.53 ± 1.13	1.55 ± 1.01	0.259
HDL-C (mmol/L)	1.18 ± 0.30	1.35 ± 0.30	1.39 ± 0.30	1.43 ± 0.31	<0.001	1.20 ± 0.31	1.39 ± 0.30	1.40 ± 0.30	1.41 ± 0.30	<0.001
LDL-C (mmol/L)	3.07 ± 0.80	2.77 ± 0.73	2.76 ± 0.71	2.66 ± 0.65	<0.001	3.01 ± 0.79	2.78 ± 0.72	2.73 ± 0.69	2.69 ± 0.71	<0.01
Systolic BP (mm/Hg)	130.49 ± 18.09	127.79 ± 17.02	125.77 ± 17.90	123.81 ± 16.58	<0.001	129.19 ± 18.96	127.96 ± 17.42	127.57 ± 16.66	124.33 ± 16.97	<0.001
Diastolic BP (mm/Hg)	81.31 ± 11.40	80.69 ± 10.84	80.40 ± 11.61	79.56 ± 10.89	0.001	81.09 ± 11.83	80.94 ± 11.38	80.74 ± 11.13	79.64 ± 10.69	0.004
Glucose (mmol/L)	6.17 ± 0.81	5.71 ± 1.08	5.54 ± 1.03	5.51 ± 1.07	<0.001	6.11 ± 0.83	5.71 ± 1.57	5.53 ± 0.72	5.50 ± 0.88	<0.001

BMI, body mass index; BP, blood pressure; HDL-C, high-density lipoprotein cholesterol; LDL-C, low-density lipoprotein cholesterol.

Values are presented as mean ± standard deviation for continuous variables and *n* (%) for categorical variables. *p*-values are from ANOVA (continuous) or chi-square tests (categorical).

Physical activity did not differ across quartiles of fruit or vegetable intake. In contrast, alcohol consumption and television watching time varied significantly across quartiles (all *p* < 0.001), although no clear monotonic trends were observed. Body weight and BMI were similar across fruit intake quartiles but differed modestly across vegetable intake quartiles. Triglyceride concentrations did not vary significantly across quartiles of either exposure.

Higher fruit and vegetable intakes were associated with higher consumption of other food groups, including soy, red meat, poultry, fish, dairy, nuts, and salted vegetables, reflecting substantial co-consumption of dietary components.

### Primary associations of fruit intake with metabolic syndrome and its components

Higher fruit intake was associated with lower prevalence and odds of MetS (Table 2). The prevalence decreased from 51.4% in the lowest quartile to 46.2% in the highest quartile. In multivariable-adjusted models, participants in the highest quartile had 18% lower odds of MetS compared with those in the lowest quartile (OR 0.82; 95% CI 0.69–0.97; *p* for trend = 0.02).

**Table 2:** Crude and adjusted odds ratios (ORs) and 95% confidence intervals (CIs) for the associations between fruit intake and metabolic syndrome and its components.

Outcome	Quartile	Prevalence (%)	Crude OR (95% CI)	*p* for trend	Adjusted OR (95% CI)^a^	*p* for trend
Metabolic syndrome	Q1 (ref)	51.4	1.00 (ref)	—	1.00 (ref)	—
	Q2	50.1	0.95 (0.82–1.10)	0.03	0.93 (0.80–1.08)	0.02
	Q3	48.6	0.89 (0.77–1.04)		0.87 (0.74–1.02)	
	Q4	46.2	**0.83 (0.71–0.98)**		**0.82 (0.69–0.97)**	
	Per 100 g/day	—	**0.93 (0.89–0.98)**	—	**0.90 (0.85–0.95)**	—
Elevated waist circumference	Q1 (ref)	58.8	1.00 (ref)	—	1.00 (ref)	—
	Q2	57.4	0.95 (0.82–1.10)	0.04	0.94 (0.81–1.09)	0.03
	Q3	56.0	0.90 (0.78–1.05)		0.88 (0.75–1.03)	
	Q4	54.2	0.85 (0.72–1.00)		**0.84 (0.70–0.99)**	
	Per 100 g/day	—	**0.94 (0.90–0.99)**	—	**0.91 (0.86–0.96)**	—
Elevated triglyceride	Q1 (ref)	33.6	1.00 (ref)	—	1.00 (ref)	—
	Q2	32.6	0.95 (0.81–1.11)	0.02	0.92 (0.78–1.08)	0.01
	Q3	30.8	0.86 (0.72–1.01)		**0.84 (0.70–0.99)**	
	Q4	28.7	**0.79 (0.66–0.95)**		**0.78 (0.64–0.94)**	
	Per 100 g/day	—	**0.91 (0.86–0.96)**	—	**0.88 (0.83–0.94)**	—
Reduced HDL-C	Q1 (ref)	54.9	1.00 (ref)	—	1.00 (ref)	—
	Q2	53.6	0.96 (0.83–1.11)	0.05	0.94 (0.81–1.09)	0.06
	Q3	52.0	0.91 (0.78–1.06)		0.90 (0.77–1.05)	
	Q4	49.8	0.85 (0.72–1.01)		0.86 (0.73–1.02)	
	Per 100 g/day	—	**0.94 (0.89–0.99)**	—	**0.93 (0.88–0.99)**	—
Elevated blood pressure	Q1 (ref)	54.9	1.00 (ref)	—	1.00 (ref)	—
	Q2	53.7	0.95 (0.82–1.10)	0.04	0.94 (0.81–1.08)	0.03
	Q3	52.1	0.90 (0.77–1.05)		0.89 (0.76–1.04)	
	Q4	50.0	**0.84 (0.71–0.99)**		0.85 (0.71–1.00)	
	Per 100 g/day	—	**0.94 (0.90–0.99)**	—	**0.92 (0.87–0.97)**	—
Elevated FBG	Q1 (ref)	52.3	1.00 (ref)	—	1.00 (ref)	—
	Q2	51.2	0.96 (0.83–1.11)	0.02	0.93 (0.80–1.07)	0.02
	Q3	49.4	0.88 (0.76–1.03)		0.86 (0.74–1.01)	
	Q4	47.3	**0.82 (0.70–0.97)**		**0.82 (0.69–0.97)**	
	Per 100 g/day	—	**0.92 (0.88–0.97)**	—	**0.89 (0.84–0.94)**	—

BP, blood pressure; FBG, fasting blood glucose; HDL-C, high-density lipoprotein cholesterol; TG, triglyceride; WC, waist circumference.

a Models were adjusted for age, sex, education, smoking, alcohol, physical activity, body mass index, sleep duration, television watching, and intakes of vegetables, red meat, poultry, fish, nuts, and soy. Bold indicates statistical significance (*p* < 0.05).

Higher fruit intake was also associated with lower odds of several MetS components, including elevated waist circumference, triglycerides, and fasting glucose, with a borderline inverse association observed for blood pressure. No statistically significant association was observed for reduced HDL-C in quartile-based analyses. Significant linear trends were observed across quartiles for these outcomes (*p* for trend ≤ 0.03).

When modeled continuously, each 100 g/day increase in fruit intake was associated with lower odds of MetS and multiple components, including waist circumference, triglycerides, blood pressure, and fasting glucose.

### Primary associations of vegetable intake with metabolic syndrome and its components

Higher vegetable intake was similarly associated with lower odds of MetS (Table 3). The prevalence declined from 52.6% in the lowest quartile to 47.5% in the highest quartile. Participants in the highest quartile had 16% lower odds of MetS than those in the lowest quartile (OR 0.84; 95% CI 0.70–0.99; *p* for trend = 0.03).

**Table 3:** Crude and adjusted odds ratios (ORs) and 95% confidence intervals (CIs) for the associations between vegetable intake and metabolic syndrome and its components.

Outcome	Quartile	Prevalence (%)	Crude OR (95% CI)	*p* for trend	Adjusted OR (95% CI)^a^	*p* for trend
Metabolic syndrome	Q1 (ref)	52.6	1.00 (ref)	—	1.00 (ref)	—
	Q2	51.0	0.94 (0.81–1.10)	0.04	0.92 (0.79–1.07)	0.03
	Q3	49.4	0.89 (0.76–1.04)		0.87 (0.74–1.02)	
	Q4	47.5	**0.83 (0.70–0.99)**		**0.84 (0.70–0.99)**	
	Per 200 g/day	—	**0.94 (0.89–0.99)**	—	**0.91 (0.85–0.98)**	—
Elevated WC	Q1 (ref)	58.5	1.00 (ref)	—	1.00 (ref)	—
	Q2	57.4	0.95 (0.82–1.10)	0.04	0.93 (0.80–1.08)	0.03
	Q3	56.0	0.90 (0.77–1.05)		0.89 (0.76–1.04)	
	Q4	54.0	**0.84 (0.71–0.99)**		0.85 (0.71–1.00)	
	Per 200 g/day	—	0.95 (0.90–1.00)	—	**0.92 (0.86–0.99)**	—
Elevated TG	Q1 (ref)	36.5	1.00 (ref)	—	1.00 (ref)	—
	Q2	35.7	0.98 (0.84–1.15)	0.07	0.96 (0.82–1.13)	0.06
	Q3	34.9	0.95 (0.81–1.11)		0.93 (0.79–1.09)	
	Q4	33.4	0.90 (0.76–1.06)		0.89 (0.74–1.06)	
	Per 200 g/day	—	0.97 (0.92–1.02)	—	0.95 (0.89–1.01)	—
Reduced HDL-C	Q1 (ref)	54.8	1.00 (ref)	—	1.00 (ref)	—
	Q2	53.8	0.97 (0.84–1.13)	0.06	0.95 (0.82–1.11)	0.07
	Q3	52.3	0.93 (0.80–1.08)		0.92 (0.79–1.07)	
	Q4	50.6	0.88 (0.75–1.03)		0.89 (0.75–1.05)	
	Per 200 g/day	—	0.95 (0.90–1.01)	—	0.94 (0.88–1.01)	—
Elevated BP	Q1 (ref)	56.8	1.00 (ref)	—	1.00 (ref)	—
	Q2	55.5	0.95 (0.82–1.11)	0.04	0.93 (0.80–1.08)	0.03
	Q3	54.1	0.91 (0.78–1.07)		0.89 (0.76–1.04)	
	Q4	52.3	0.85 (0.72–1.00)		0.85 (0.71–1.00)	
	Per 200 g/day	—	**0.94 (0.89–0.99)**	—	**0.92 (0.86–0.99)**	—
Elevated FBG	Q1 (ref)	53.7	1.00 (ref)	—	1.00 (ref)	—
	Q2	52.5	0.95 (0.82–1.11)	0.04	0.94 (0.81–1.10)	0.03
	Q3	51.0	0.91 (0.78–1.06)		0.90 (0.77–1.05)	
	Q4	49.4	0.86 (0.73–1.01)		0.86 (0.72–1.01)	
	Per 200 g/day	—	0.95 (0.90–1.00)	—	0.93 (0.86–1.00)	—

BP, blood pressure; FBG, fasting blood glucose; HDL-C, high-density lipoprotein cholesterol; TG, triglyceride; WC, waist circumference.

a Models were adjusted for age, sex, education, smoking, alcohol, physical activity, body mass index, sleep duration, television watching, and intakes of fruits, red meat, poultry, fish, nuts, and soy. Bold indicates statistical significance (*p* < 0.05).

In analyses of individual components, higher vegetable intake showed inverse but generally weaker associations. Borderline inverse associations were observed for waist circumference, blood pressure, and fasting glucose, whereas associations with triglycerides and HDL-C were not statistically significant.

Continuous analyses showed that each 200 g/day increase in vegetable intake was associated with lower odds of MetS, waist circumference, blood pressure, and fasting glucose, while associations with triglycerides and HDL-C remained non-significant.

### Restricted cubic spline analyses

**Figure 1:**
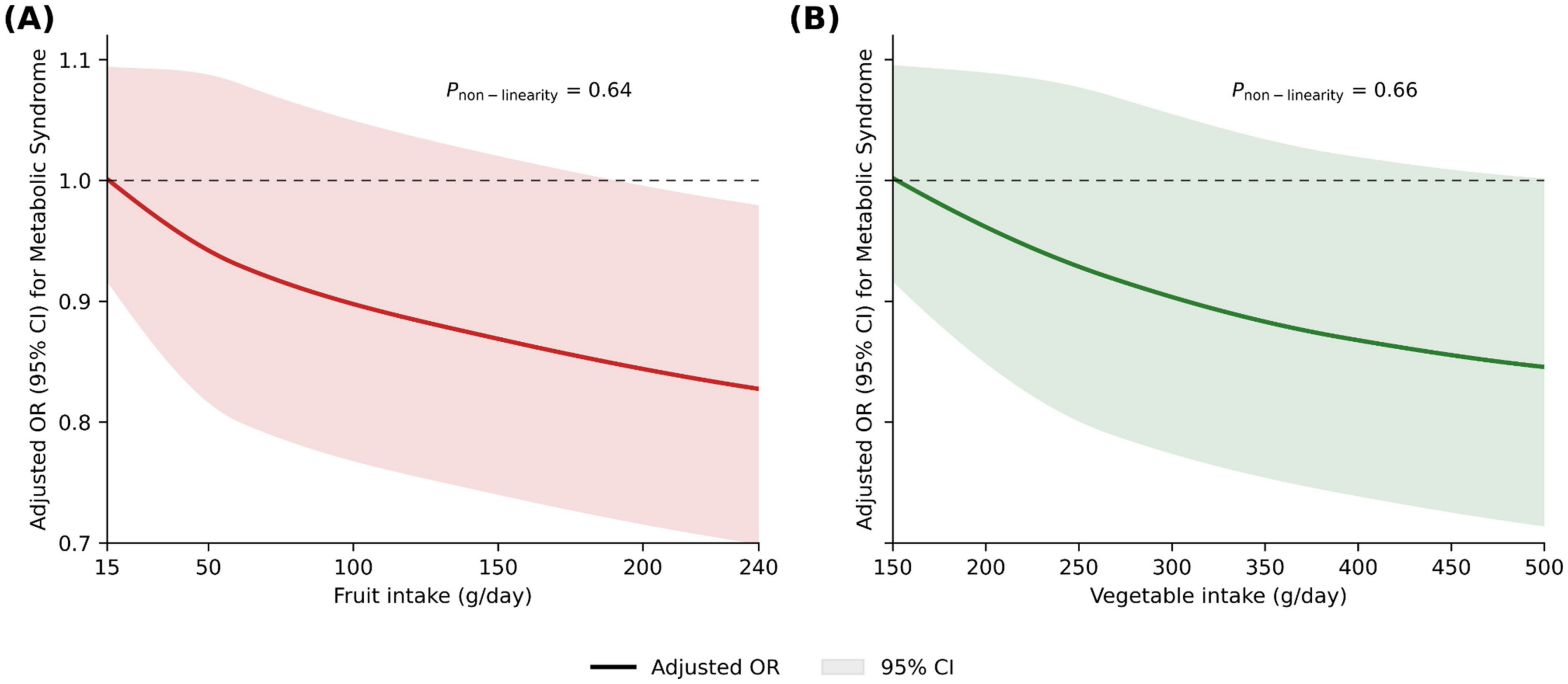
Restricted cubic spline analyses of the associations of **(A)** fruit and **(B)** vegetable intakes with metabolic syndrome. Solid lines indicate adjusted odds ratios (ORs), and shaded areas indicate 95% confidence intervals (CIs). Models were adjusted for the same covariates as the primary analyses. No statistically significant non-linearity was detected for fruit intake (*p* = 0.64) or vegetable intake (*p* = 0.66).

Restricted cubic spline analyses were conducted to examine potential non-linear associations between fruit and vegetable intakes and MetS (Figures 1A,B). The spline curves were generally consistent with inverse associations between fruit and vegetable intakes and MetS across the observed intake ranges. However, there was no statistically significant evidence of non-linearity for fruit intake (*p* for non-linearity = 0.64) or vegetable intake (*p* for non-linearity = 0.66).

### Subgroup analyses

Inverse associations between fruit intake and MetS were consistently observed across subgroups defined by age, sex, BMI, and smoking status (Table 4). Associations were generally stronger among younger participants, women, individuals with BMI ≥ 25 kg/m^2^, and never smokers, although no statistically significant interactions were detected (all *p* for interaction > 0.08). Similar patterns were observed for individual MetS components.

**Table 4:** Adjusted odds ratios (ORs) and 95% confidence intervals (CIs) for the associations between fruit or vegetable intake and metabolic syndrome and its components by age, sex, body mass index, and smoking status.

Subgroup	Metabolic syndrome OR (95% CI)	*p* for interaction	Elevated WC OR (95% CI)	*p* for interaction	Elevated TG OR (95% CI)	*p* for interaction	Reduced HDL-C OR (95% CI)	*p* for interaction	Elevated BP OR (95% CI)	*p* for interaction	Elevated FBG OR (95% CI)	*p* for interaction
Fruits (per 100 g/day)												
*Age*												
Age <55	**0.90 (0.82–0.97)**		**0.89 (0.81–0.97)**		**0.88 (0.79–0.94)**		**0.87 (0.78–0.95)**		**0.88 (0.80–0.94)**		**0.87 (0.77–0.95)**	
Age ≥55	0.94 (0.85–1.04)	0.21	0.93 (0.85–1.03)	0.18	**0.92 (0.83–0.98)**	0.12	0.95 (0.87–1.04)	0.15	0.93 (0.84–1.02)	0.19	0.93 (0.84–1.01)	0.16
*Sex*												
Men	0.95 (0.88–1.04)		0.93 (0.85–1.00)		**0.91 (0.82–0.98)**		**0.92 (0.83–0.98)**		**0.92 (0.82–0.98)**		0.92 (0.83–1.00)	
Women	**0.90 (0.82–0.96)**	0.14	**0.88 (0.81–0.96)**	0.13	**0.87 (0.80–0.94)**	0.11	**0.86 (0.78–0.94)**	0.08	**0.89 (0.82–0.96)**	0.10	**0.88 (0.81–0.95)**	0.12
*BMI*												
BMI <25	0.94 (0.86–1.01)		0.91 (0.83–1.00)		**0.90 (0.82–0.98)**		**0.91 (0.83–0.99)**		0.92 (0.83–1.00)		0.93 (0.84–1.01)	
BMI ≥25	**0.91 (0.83–0.99)**	0.19	**0.89 (0.81–0.97)**	0.17	**0.87 (0.79–0.94)**	0.14	**0.86 (0.78–0.94)**	0.10	**0.88 (0.81–0.95)**	0.11	**0.89 (0.82–0.96)**	0.13
*Smoking*												
Never smokers	**0.89 (0.82–0.96)**		**0.87 (0.80–0.94)**		**0.86 (0.78–0.94)**		**0.85 (0.77–0.92)**		**0.87 (0.80–0.94)**		**0.88 (0.81–0.95)**	
Former/Current smokers	0.94 (0.86–1.03)	0.16	**0.91 (0.83–0.99)**	0.15	**0.90 (0.82–0.98)**	0.12	**0.91 (0.83–0.99)**	0.14	**0.92 (0.84–0.99)**	0.13	0.92 (0.84–1.00)	0.11
Vegetables (per 200 g/day)												
*Age*												
Age <55	**0.92 (0.84–0.99)**		0.93 (0.86–1.00)		0.94 (0.87–1.03)		0.94 (0.87–1.04)		0.95 (0.88–1.03)		0.94 (0.87–1.02)	
Age ≥55	0.99 (0.93–1.08)	0.23	0.99 (0.92–1.07)	0.20	0.99 (0.92–1.06)	0.17	0.98 (0.89–1.05)	0.21	0.95 (0.85–1.02)	0.19	0.96 (0.89–1.02)	0.18
*Sex*												
Men	0.96 (0.88–1.04)		0.95 (0.87–1.02)		0.93 (0.85–1.01)		0.94 (0.86–1.02)		0.95 (0.87–1.03)		0.95 (0.87–1.03)	
Women	**0.92 (0.84–0.99)**	0.12	**0.91 (0.84–0.98)**	0.11	0.93 (0.86–1.01)	0.10	0.94 (0.87–1.01)	0.13	0.93 (0.86–1.00)	0.10	0.94 (0.87–1.01)	0.09
*BMI*												
BMI <25	0.97 (0.89–1.05)		0.96 (0.88–1.03)		0.95 (0.87–1.03)		0.96 (0.88–1.04)		0.97 (0.89–1.04)		0.96 (0.88–1.05)	
BMI ≥25	0.94 (0.86–1.02)	0.24	0.93 (0.85–1.01)	0.21	**0.92 (0.84–0.99)**	0.19	0.93 (0.85–1.00)	0.22	0.94 (0.86–1.01)	0.20	0.95 (0.87–1.02)	0.23
*Smoking*												
Never smokers	0.94 (0.87–1.01)		**0.92 (0.85–0.99)**		**0.91 (0.83–0.98)**		**0.92 (0.84–0.99)**		0.93 (0.85–1.00)		0.94 (0.86–1.01)	
Former/current smokers	0.97 (0.89–1.05)	0.15	0.95 (0.88–1.03)	0.13	0.96 (0.88–1.04)	0.11	0.96 (0.88–1.05)	0.12	0.96 (0.89–1.03)	0.11	0.96 (0.89–1.04)	0.13

BP, blood pressure; FBG, fasting blood glucose; HDL-C, high-density lipoprotein cholesterol; TG, triglyceride; WC, waist circumference.

ORs and 95% CIs were estimated using logistic regression models. Models were adjusted for age, sex, education, smoking, alcohol consumption, physical activity, body mass index, sleep duration, television watching, and intakes of red meat, poultry, fish, nuts, soy, and dairy, with mutual adjustment for fruit and vegetable intake; the stratifying variable was excluded from the corresponding model. *p* for interaction was derived from a Wald test for the multiplicative interaction term between fruit (per 100 g/day) or vegetable (per 200 g/day) intake and the stratifying variable in fully adjusted logistic regression models. Bold indicates statistical significance (*p* < 0.05).

For vegetable intake, inverse associations with MetS were also generally consistent across subgroups, with somewhat stronger associations among younger participants and women. Associations with individual components were directionally similar but less consistent. No statistically significant interactions were detected (all *p* for interaction >0.09).

### Joint associations of fruit or vegetable intake with other food groups

In joint analyses, higher fruit intake combined with lower red meat intake was consistently associated with lower odds of MetS and all of its individual components (Supplementary Table 1). Similar inverse associations were observed for combinations of higher fruit intake with lower intake of other food groups, although associations with waist circumference, blood pressure, and fasting glucose were generally weaker and often borderline statistically significant.

In the joint analyses, combinations involving higher vegetable intake showed more consistent inverse associations across outcomes (Supplementary Table 2). Higher vegetable intake, combined with lower red meat intake, was associated with lower odds of MetS and of all its individual components. Combinations of higher vegetable intake with lower intake of other food groups were also associated with lower odds of MetS and several components, particularly triglycerides, HDL-C, and waist circumference, while associations with blood pressure and fasting glucose were generally weaker. Conversely, combinations characterized by lower fruit or vegetable intake and higher red meat intake were generally associated with higher odds of MetS and several components, or showed no clear association for some outcomes.

## Discussion

In this community-based cross-sectional study of Chinese adults, higher habitual intakes of fruits and vegetables were associated with lower odds of metabolic syndrome (MetS). Participants in the highest quartile of fruit and vegetable intakes had 18 and 16% lower odds of MetS, respectively, compared with those in the lowest quartile, with similar inverse associations observed in continuous analyses. Higher fruit intake was consistently associated with lower odds across multiple MetS components, including central adiposity, triglycerides, blood pressure, and fasting glucose, whereas associations for vegetable intake were generally weaker and more component-specific, particularly for central adiposity, blood pressure, and fasting glucose. These associations were broadly consistent across population subgroups, with no statistically significant interactions observed.

Our findings are consistent with prior observational studies and meta-analyses, largely conducted in Western populations, reporting inverse associations between fruit and vegetable intakes and MetS (15, 16). However, evidence from Chinese populations has been limited and inconsistent (17–20). Many previous studies examined fruit and vegetable intakes as secondary exposures and focused primarily on MetS as a composite outcome. In contrast, the present study treated fruit and vegetable intakes as primary exposures and examined both overall MetS and its individual components. Notably, the associations differed by component, with fruit intake showing more consistent inverse associations than vegetable intake, particularly for fasting glucose and triglycerides. Although the mechanisms underlying these differences cannot be established in the present cross-sectional study, they may partly reflect variation in consumption patterns, nutrient bioavailability, and preparation methods. Fruits and vegetables provide dietary fiber and phytochemicals that have been associated with favorable cardiometabolic effects in clinical trials, including improvements in fasting glucose, insulin resistance, blood pressure, and lipid metabolism (21, 22). Preparation practices may also partly influence these associations. Fruits are typically consumed raw, whereas vegetables in Chinese diets are often cooked or prepared with added oils, sauces, or salt. Thermal processing may reduce concentrations of heat-sensitive compounds such as glucosinolates and vitamin C (23), while added oils or sodium may introduce additional dietary exposures that could attenuate or obscure inverse associations. These culinary differences may help explain why vegetable intake showed weaker or more selective associations in the present study than fruit intake, and why these associations differed from findings in some Western populations, where raw vegetable consumption may be more common. Notably, restricted cubic spline analyses showed inverse associations between fruit and vegetable intakes and prevalent MetS, with no statistically significant evidence of non-linearity. The observed intake distributions encompassed the recommendations in the current Chinese Dietary Guidelines for fresh fruit (200–350 g/day) and vegetables (300–500 g/day) (9). Within these intake ranges, the spline analyses did not identify a clear threshold, inflection point, or plateau in the associations with prevalent MetS. Although these findings do not establish optimal intake levels or causal effects, they suggest that the observed associations were not confined to a specific intake level within the ranges studied.

The joint analyses provide additional insight into how associations of fruit and vegetable intakes with MetS may vary across co-consumption patterns of other major food groups. Higher fruit or vegetable intake combined with lower red meat intake was consistently associated with lower odds of MetS and its individual components, whereas combinations characterized by lower fruit or vegetable intake and higher red meat intake were generally associated with higher odds of MetS and several of its components or showed no clear association. Although these analyses were exploratory and did not establish causal interaction, several biological mechanisms may plausibly contribute to these patterns. Lower red meat intake may reduce exposure to saturated fat and heme iron, which have been linked to elevated blood cholesterol, oxidative stress, lipid peroxidation, and insulin resistance (24). In contrast, dietary patterns characterized by higher fruit and vegetable intake may reflect broader dietary behaviors associated with more favorable metabolic profiles. Combinations of higher fruit or vegetable intake with greater consumption of fish, dairy, or soy were also associated with lower odds of MetS and several components, broadly consistent with previous analyses from this study population reporting associations of these food groups with cardiometabolic outcomes (12–14). Overall, these findings suggest that associations between fruit and vegetable intakes and metabolic health may depend, in part, on the broader dietary context in which these foods are consumed rather than on isolated food groups alone. This interpretation is consistent with findings from dietary pattern studies in Chinese populations, which have reported lower MetS prevalence with plant-forward or mixed plant–aquatic patterns and higher prevalence with meat-rich patterns (25, 26).

Several limitations should be considered. First, temporal direction cannot be established due to the cross-sectional design. Reverse causation is possible, whereby individuals diagnosed with hypertension, dyslipidemia, or impaired glucose regulation may have subsequently increased fruit or vegetable consumption following medical advice or health concerns. Such behavioral modification could attenuate or otherwise influence the observed associations, particularly for vegetables, where effect sizes were modest, and several associations were borderline statistically significant. Accordingly, the observed ORs should be interpreted as prevalence associations rather than estimates of causal effect. Second, dietary intake was assessed using an FFQ and is therefore subject to recall error and reporting bias. The lack of detailed information on total energy intake, food subtypes, and preparation methods may have introduced measurement error and limited the ability to account for overall diet quality. The relatively limited granularity of the five-category FFQ may therefore have introduced non-differential exposure misclassification, which would likely bias associations toward the null. Residual confounding by total energy intake, therefore, cannot be excluded. In addition, cooking methods may represent an important source of residual confounding in this population. In Chinese dietary practice, vegetables are frequently stir-fried or prepared with added oils, sauces, or salt, which may alter nutrient bioavailability and introduce additional metabolic exposures, including sodium and cooking fats. Since preparation methods were not captured in the FFQ, the observed associations may partly reflect the combined effects of vegetables and their preparation practices rather than vegetable intake alone. Third, residual confounding cannot be ruled out, as fruit and vegetable intakes are closely correlated with other health-related behaviors and aspects of diet that may not be fully captured by measured covariates. Although joint analyses accounted for co-consumption of major food groups, unmeasured dietary or nutrient-level factors may still contribute to the observed associations. Fourth, participants were recruited from urban communities in Suzhou Industrial Park, an economically developed region with relatively high healthcare access and health literacy. Dietary behaviors and cardiometabolic risk profiles in this population may differ from those in rural or lower-income regions of China, potentially limiting generalizability. Finally, the joint analyses were exploratory and descriptive and do not constitute formal tests of interaction or comprehensive dietary pattern analyses; therefore, they should be interpreted with caution and not as evidence of biological interaction or causal synergy.

## Conclusion

In this community-based sample of Chinese adults, higher fruit and vegetable intakes were associated with lower odds of MetS, with fruit showing broader inverse associations across individual components than vegetables. Joint analyses suggested that these associations varied according to co-consumption patterns of other major food groups, particularly red meat intake, highlighting the importance of considering overall dietary context when interpreting associations between specific food groups and metabolic health.

## Data Availability

The raw data supporting the conclusions of this article will be made available by the authors, without undue reservation.

## References

[1] Alberti KG Eckel RH Grundy SM Zimmet PZ Cleeman JI Donato KA . Harmonizing the metabolic syndrome: a joint interim statement of the international diabetes federation task force on epidemiology and prevention; National Heart, Lung, and Blood Institute; American Heart Association; world heart federation; international atherosclerosis society; and International Association for the Study of obesity. Circulation. (2009) 120:1640–5. doi: 10.1161/circulationaha.109.192644, 19805654

[2] Saklayen MG. The global epidemic of the metabolic syndrome. Curr Hypertens Rep. (2018) 20:12. doi: 10.1007/s11906-018-0812-z, 29480368 PMC5866840

[3] Ranasinghe P Mathangasinghe Y Jayawardena R Hills AP Misra A. Prevalence and trends of metabolic syndrome among adults in the Asia-Pacific region: a systematic review. BMC Public Health. (2017) 17:101. doi: 10.1186/s12889-017-4041-1, 28109251 PMC5251315

[4] Huang J Huang JLW Withers M Xu X Li S Wang Y . Prevalence of metabolic syndrome in Chinese women and men: a systematic review and meta-analysis of data from 734,511 individuals. Lancet. (2018) 392:S14

[5] Pérez-Martínez P Mikhailidis DP Athyros VG Bulló M Couture P Covas MI . Lifestyle recommendations for the prevention and management of metabolic syndrome: an international panel recommendation. Nutr Rev. (2017) 75:307–26. doi: 10.1093/nutrit/nux014, 28521334 PMC5914407

[6] Wallace TC Bailey RL Blumberg JB Burton-Freeman B Chen CO Crowe-White KM . Fruits, vegetables, and health: a comprehensive narrative, umbrella review of the science and recommendations for enhanced public policy to improve intake. Crit Rev Food Sci Nutr. (2020) 60:2174–211. doi: 10.1080/10408398.2019.1632258, 31267783

[7] Devirgiliis C Guberti E Mistura L Raffo A. Effect of fruit and vegetable consumption on human health: an update of the literature. Foods. (2024) 13:3149. doi: 10.3390/foods13193149, 39410184 PMC11475733

[8] Slavin JL Lloyd B. Health benefits of fruits and vegetables. Adv Nutr. (2012) 3:506–16. doi: 10.3945/an.112.002154, 22797986 PMC3649719

[9] Chinese Nutrition Society. Dietary Guidelines for Chinese Residents (2022). Beijing: People's Medical Publishing House (2022).

[10] Li YC Jiang B Zhang M Huang ZJ Deng Q Zhou MG . Vegetable and fruit consumption among Chinese adults and associated factors: a nationally representative study of 170,847 adults. Biomed Environ Sci. (2017) 30:863–74. doi: 10.3967/bes2017.117, 29335056

[11] Huang L Wang Z Wang H Zhao L Jiang H Zhang B . Nutrition transition and related health challenges over decades in China. Eur J Clin Nutr. (2021) 75:247–52. doi: 10.1038/s41430-020-0674-8, 32620907

[12] Hidayat K Yu LG Yang JR Zhang XY Zhou H Shi YJ . The association between milk consumption and the metabolic syndrome: a cross-sectional study of the residents of Suzhou, China and a meta-analysis. Br J Nutr. (2020) 123:1013–23. doi: 10.1017/S0007114520000227, 31964442

[13] Hidayat K Zhu WZ Peng SM Ren JJ Lu ML Wang HP . The association between meat consumption and the metabolic syndrome: a cross-sectional study and meta-analysis. Br J Nutr. (2022) 127:1467–81. doi: 10.1017/S0007114521002452, 34420528

[14] Hidayat K Huang YH Qian XY Chen XF Yu LG Zhou H . The association between soy consumption and metabolic syndrome in Chinese adults: a cross-sectional study. Front Nutr. (2025) 12:1637413. doi: 10.3389/fnut.2025.1637413, 40896186 PMC12394039

[15] Lee M Lim M Kim J. Fruit and vegetable consumption and the metabolic syndrome: a systematic review and dose-response meta-analysis. Br J Nutr. (2019) 122:723–33. doi: 10.1017/S000711451900165X, 31514758

[16] Tian Y Su L Wang J Duan X Jiang X. Fruit and vegetable consumption and risk of the metabolic syndrome: a meta-analysis. Public Health Nutr. (2018) 21:756–65. doi: 10.1017/S136898001700310X, 29151369 PMC10260986

[17] Lin YH Chang HT Tseng YH Lin MH Chen YC Yang HW . Characteristics and health behavior of newly developed metabolic syndrome among community-dwelling elderly in Taiwan. Int J Gerontol. (2013) 7:90–6. doi: 10.1016/j.ijge.2012.07.003

[18] Guo H Gao X Ma R Liu J Ding Y Zhang M . Prevalence of metabolic syndrome and its associated factors among multi-ethnic adults in rural areas in Xinjiang, China. Sci Rep. (2017) 7:17643. doi: 10.1038/s41598-017-17870-5, 29247195 PMC5732195

[19] Li Y Zhao L Yu D Wang Z Ding G. Metabolic syndrome prevalence and its risk factors among adults in China: a nationally representative cross-sectional study. PLoS One. (2018) 13:e0199293. doi: 10.1371/journal.pone.0199293, 29920555 PMC6007893

[20] Lin WT Lee CY Tsai S Huang HL Wu PW Chin YT . Clustering of metabolic risk components and associated lifestyle factors: a nationwide adolescent study in Taiwan. Nutrients. (2019) 11:584. doi: 10.3390/nu11030584, 30857325 PMC6471895

[21] Fu L Zhang G Qian S Zhang Q Tan M. Associations between dietary fiber intake and cardiovascular risk factors: an umbrella review of meta-analyses of randomized controlled trials. Front Nutr. (2022) 9:972399. doi: 10.3389/fnut.2022.972399, 36172520 PMC9511151

[22] Arshad MT Ali MKM Maqsood S Ikram A Hossain MS Aljameel AI . Dietary phytochemicals in cardiovascular disease prevention and management: a comprehensive review. Food Sci Nutr. (2025) 13:e70872. doi: 10.1002/fsn3.70872, 40909258 PMC12406175

[23] Yuan GF Sun B Yuan J Wang QM. Effects of different cooking methods on health-promoting compounds of broccoli. J Zhejiang Univ Sci B. (2009) 10:580–8. doi: 10.1631/jzus.B0920051, 19650196 PMC2722699

[24] Hidayat K Chen JS Wang HP Wang TC Liu YJ Zhang XY . Is replacing red meat with other protein sources associated with lower risks of coronary heart disease and all-cause mortality? A meta-analysis of prospective studies. Nutr Rev. (2022) 80:1959–73. doi: 10.1093/nutrit/nuac017, 35380734

[25] Wei ZY Liu JJ Zhan XM Feng HM Zhang YY. Dietary patterns and the risk of metabolic syndrome in Chinese adults: a population-based cross-sectional study. Public Health Nutr. (2018) 21:2409–16. doi: 10.1017/S1368980018001088, 29717687 PMC6137368

[26] Wang Y Dai Y Tian T Zhang J Xie W Pan D . The effects of dietary pattern on metabolic syndrome in Jiangsu province of China: based on a nutrition and diet investigation project in Jiangsu province. Nutrients. (2021) 13:4451. doi: 10.3390/nu13124451, 34960003 PMC8708757

