# Supplementary material for: Fruit and vegetable consumption and metabolic syndrome in Chinese adults: a cross-sectional study
Source: Front Nutr. 2026 Jun 30;13:1852298. doi: 10.3389/fnut.2026.1852298 (PMC13364958; doi:10.3389/fnut.2026.1852298)
Supplement: Supplementary file 1 [file Table_1.docx]

**Supplementary Table 1** Adjusted odds ratios (ORs) and 95% confidence intervals (CIs) for the joint associations of fruit intake with various food groups in relation to metabolic syndrome and its components

| **Outcome** | **High fruits + Low red meat** | **Low fruits + High red meat** | **High fruits + High red meat** | **High fruits + Low poultry** | **Low fruits + High poultry** | **High fruits + High poultry** | **High fruits + Low fish** | **Low fruits + High fish** | **High fruits + High fish** | **High fruits + Low soy** | **Low fruits + High soy** | **High fruits + High soy** | **High fruits + No nuts** | **Low fruits + Nuts** | **High fruits + Nuts** | **High fruits + No dairy** | **Low fruits + Dairy** | **High fruits + Dairy** |
| --- | --- | --- | --- | --- | --- | --- | --- | --- | --- | --- | --- | --- | --- | --- | --- | --- | --- | --- |
| **Metabolic syndrome** | **0.75 (0.64–0.88)** | 1.10 (0.96–1.27) | 0.94 (0.81–1.10) | 0.79 (0.66–0.95) | 1.01 (0.87–1.17) | 0.92 (0.78–1.07) | **0.78 (0.65–0.93)** | 0.90 (0.76–1.06) | **0.83 (0.70–0.98)** | **0.76 (0.63–0.91)** | 0.85 (0.71–1.01) | **0.78 (0.65–0.94)** | **0.82 (0.68–0.99)** | 0.90 (0.76–1.07) | 0.86 (0.72–1.02) | **0.77 (0.64–0.92)** | 0.86 (0.72–1.03) | **0.81 (0.68–0.97)** |
| **Elevated BP** | **0.77 (0.65–0.91)** | 1.08 (0.94–1.25) | 0.96 (0.82–1.13) | **0.76 (0.62–0.93)** | 1.00 (0.85–1.17) | 0.93 (0.78–1.10) | **0.80 (0.66–0.96)** | 0.91 (0.77–1.08) | 0.85 (0.71–1.02) | **0.78 (0.64–0.94)** | 0.88 (0.74–1.05) | **0.80 (0.66–0.96)** | 0.84 (0.70–1.00) | 0.91 (0.76–1.09) | 0.88 (0.74–1.05) | **0.79 (0.65–0.96)** | 0.87 (0.72–1.04) | 0.83 (0.69–1.00) |
| **Elevated TG** | **0.73 (0.60–0.88)** | 1.07 (0.92–1.25) | 0.93 (0.78–1.10) | **0.72 (0.58–0.88)** | 1.03 (0.87–1.20) | 0.90 (0.76–1.07) | **0.75 (0.61–0.91)** | 0.87 (0.72–1.04) | 0.84 (0.70–1.00) | **0.74 (0.60–0.90)** | 0.84 (0.69–1.02) | **0.79 (0.65–0.96)** | 0.80 (0.66–0.97) | 0.87 (0.73–1.04) | 0.84 (0.70–1.00) | **0.76 (0.62–0.93)** | 0.86 (0.72–1.03) | **0.82 (0.68–0.98)** |
| **Reduced HDL-C** | **0.78 (0.66–0.92)** | 1.05 (0.91–1.21) | 0.94 (0.80–1.10) | **0.75 (0.62–0.91)** | 0.99 (0.85–1.15) | 0.91 (0.77–1.07) | **0.80 (0.66–0.96)** | 0.89 (0.75–1.06) | 0.85 (0.71–1.01) | **0.77 (0.64–0.94)** | 0.86 (0.72–1.03) | **0.80 (0.66–0.97)** | 0.82 (0.68–0.99) | 0.90 (0.76–1.07) | 0.86 (0.72–1.03) | **0.78 (0.65–0.95)** | 0.87 (0.72–1.04) | 0.83 (0.69–1.00) |
| **Elevated waist circumference** | **0.81 (0.68–0.96)** | 1.13 (0.97–1.32) | 0.96 (0.81–1.12) | **0.79 (0.65–0.95)** | 1.05 (0.89–1.22) | 0.92 (0.78–1.09) | 0.83 (0.69–1.00) | 0.91 (0.77–1.08) | 0.86 (0.72–1.02) | **0.80 (0.66–0.96)** | 0.87 (0.72–1.04) | **0.82 (0.68–0.99)** | 0.83 (0.69–1.00) | 0.91 (0.76–1.08) | 0.87 (0.72–1.04) | **0.81 (0.67–0.98)** | 0.88 (0.74–1.05) | 0.84 (0.70–1.01) |
| **Elevated FBG** | **0.84 (0.71–0.99)** | 1.02 (0.88–1.19) | 0.95 (0.81–1.12) | **0.82 (0.68–0.98)** | 1.00 (0.85–1.17) | 0.92 (0.78–1.09) | 0.85 (0.71–1.02) | 0.91 (0.77–1.08) | 0.86 (0.72–1.02) | **0.82 (0.68–0.99)** | 0.88 (0.74–1.05) | 0.83 (0.69–1.00) | 0.84 (0.70–1.01) | 0.91 (0.76–1.08) | 0.88 (0.74–1.05) | 0.83 (0.69–1.00) | 0.89 (0.74–1.06) | 0.85 (0.71–1.02) |

BP, blood pressure; FBG, fasting blood glucose; HDL-C, high-density lipoprotein cholesterol; TG, triglyceride; WC, waist circumference

ORs and 95% CIs were estimated using logistic regression models. Models were adjusted for age, sex, education, smoking, alcohol, physical activity, body mass index, sleep duration, television watching, and intakes of vegetables, red meat, poultry, fish, nuts, soy, and dairy; the food group used in the joint exposure was excluded from the corresponding model. Bold indicates statistical significance (*P* < 0.05). Reference group: low fruit intake combined with low intake of the corresponding food group; for nuts and dairy analyses, the reference group was low fruit intake combined with no consumption of nuts or dairy.

**Supplementary Table 2** Adjusted odds ratios (ORs) and 95% confidence intervals (CIs) for the joint associations of vegetable intake with various food groups in relation to metabolic syndrome and its components

| **Outcomes** | **High vegetables + Low red meat** | **Low vegetables + High red meat** | **High vegetables + High red meat** | **High vegetables + Low poultry** | **Low vegetables + High poultry** | **High vegetables + High poultry** | **High vegetables + Low fish** | **Low vegetables + High fish** | **High vegetables + High fish** | **High vegetables + Low soy** | **Low vegetables + High soy** | **High vegetables + High soy** | **High vegetables + No nuts** | **Low vegetables + Nuts** | **High vegetables + Nuts** | **High vegetables + No dairy** | **Low vegetables + Dairy** | **High vegetables + Dairy** |
| --- | --- | --- | --- | --- | --- | --- | --- | --- | --- | --- | --- | --- | --- | --- | --- | --- | --- | --- |
| **Metabolic Syndrome** | **0.73 (0.61–0.87)** | 1.14 (0.97–1.34) | 0.92 (0.77–1.09) | **0.75 (0.62–0.91)** | 0.88 (0.74–1.05) | 0.86 (0.72–1.03) | **0.73 (0.60–0.89)** | 0.86 (0.72–1.04) | **0.81 (0.67–0.97)** | **0.74 (0.61–0.90)** | 0.88 (0.72–1.06) | **0.81 (0.67–0.97)** | **0.76 (0.63–0.93)** | 0.87 (0.72–1.05) | **0.80 (0.66–0.96)** | **0.78 (0.64–0.94)** | 0.86 (0.71–1.04) | **0.77 (0.63–0.93)** |
| **Elevated BP** | **0.75 (0.63–0.89)** | 1.12 (0.95–1.33) | 0.91 (0.76–1.08) | **0.76 (0.62–0.92)** | 0.89 (0.74–1.07) | 0.87 (0.73–1.04) | **0.75 (0.62–0.91)** | 0.87 (0.73–1.05) | **0.84 (0.69–1.01)** | **0.76 (0.62–0.93)** | 0.89 (0.73–1.07) | **0.82 (0.68–0.99)** | **0.77 (0.64–0.94)** | 0.88 (0.73–1.06) | **0.82 (0.68–0.99)** | **0.79 (0.65–0.96)** | 0.88 (0.73–1.07) | **0.78 (0.64–0.95)** |
| **Elevated TG** | **0.70 (0.57–0.86)** | 1.17 (0.98–1.40) | 0.91 (0.77–1.08) | **0.70 (0.56–0.87)** | 0.86 (0.71–1.04) | 0.83 (0.69–1.01) | **0.71 (0.57–0.88)** | 0.84 (0.69–1.01) | **0.78 (0.64–0.94)** | **0.70 (0.57–0.87)** | 0.85 (0.69–1.04) | **0.78 (0.64–0.94)** | **0.71 (0.57–0.88)** | 0.84 (0.69–1.03) | **0.76 (0.62–0.93)** | **0.73 (0.59–0.90)** | 0.84 (0.69–1.03) | **0.75 (0.61–0.91)** |
| **Reduced HDL-C** | **0.75 (0.62–0.91)** | 1.10 (0.93–1.29) | 0.88 (0.74–1.05) | **0.74 (0.60–0.90)** | 0.87 (0.73–1.05) | 0.85 (0.71–1.02) | **0.75 (0.61–0.91)** | 0.84 (0.70–1.02) | **0.79 (0.65–0.95)** | **0.75 (0.61–0.92)** | 0.86 (0.70–1.05) | **0.80 (0.66–0.96)** | **0.75 (0.61–0.92)** | 0.85 (0.70–1.03) | **0.78 (0.64–0.95)** | **0.76 (0.62–0.93)** | 0.84 (0.69–1.02) | **0.76 (0.62–0.93)** |
| **Elevated WC** | **0.78 (0.65–0.94)** | 1.15 (0.96–1.38) | 0.92 (0.78–1.09) | **0.78 (0.64–0.94)** | 0.88 (0.74–1.06) | 0.85 (0.71–1.02) | **0.79 (0.65–0.96)** | 0.86 (0.72–1.04) | **0.81 (0.67–0.98)** | **0.77 (0.63–0.94)** | 0.87 (0.72–1.06) | **0.81 (0.67–0.98)** | **0.78 (0.64–0.94)** | 0.86 (0.71–1.04) | **0.80 (0.66–0.97)** | **0.79 (0.65–0.96)** | 0.85 (0.70–1.03) | **0.78 (0.64–0.94)** |
| **Elevated FBG** | **0.78 (0.65–0.94)** | 1.11 (0.93–1.34) | 0.91 (0.76–1.08) | **0.79 (0.65–0.96)** | 0.90 (0.75–1.09) | 0.87 (0.72–1.04) | **0.78 (0.64–0.95)** | 0.85 (0.71–1.03) | **0.81 (0.67–0.98)** | **0.78 (0.64–0.95)** | 0.88 (0.72–1.07) | **0.82 (0.68–0.99)** | **0.79 (0.65–0.96)** | 0.88 (0.73–1.06) | **0.81 (0.67–0.98)** | **0.80 (0.66–0.97)** | 0.87 (0.72–1.05) | **0.79 (0.65–0.96)** |

BP, blood pressure; FBG, fasting blood glucose; HDL-C, high-density lipoprotein cholesterol; TG, triglyceride; WC, waist circumference

ORs and 95% CIs were estimated using logistic regression models. Models were adjusted for age, sex, education, smoking, alcohol, physical activity, body mass index, sleep duration, television watching, and intakes of fruits, red meat, poultry, fish, nuts, soy, and dairy; the food group used in the joint exposure was excluded from the corresponding model. Bold indicates statistical significance (*P* < 0.05). Reference group: low vegetable intake combined with low intake of the corresponding food group; for nuts and dairy analyses, the reference group was low vegetable intake combined with no consumption of nuts or dairy.
